# Transcatheter Aortic Valve Replacement for Treating Native Aortic Regurgitation: Ready for Prime Time?

**DOI:** 10.1016/j.shj.2025.100725

**Published:** 2025-08-19

**Authors:** Raviteja R. Guddeti, Nadia El-Hangouche, Geoffrey Answini, Dean Kereiakes, Santiago Garcia

**Affiliations:** a Division of Interventional Cardiology, The Carl and Edyth Lindner Research Center at the Christ Hospital, Cincinnati, Ohio, USA; b Division of Cardiology, The Carl and Edyth Lindner Research Center at the Christ Hospital, Cincinnati, Ohio, USA; c Division of Cardiac Surgery, The Carl and Edyth Lindner Research Center at the Christ Hospital, Cincinnati, Ohio, USA

**Keywords:** Aortic regurgitation, Cardiac magnetic resonance imaging, Echocardiography, Surgical aortic valve replacement, Transcatheter aortic valve replacement, Transcatheter heart valve

## Abstract

Untreated clinically significant aortic regurgitation (AR) is frequently seen in the general population and is associated with worse outcomes, including higher mortality. Surgical aortic valve replacement is currently the treatment of choice for severe AR. However, a significant proportion of these patients are not good surgical candidates due to advanced age, frailty, and underlying comorbidities, prompting the need for transcatheter options. Current guidelines do not recommend transcatheter aortic valve replacement (TAVR) for severe AR with commercially available transcatheter heart valves (THVs). Off-label use of commercial TAVR devices has been associated with lower procedural success, increased complications, mainly valve embolization and residual AR, and poor clinical outcomes. The suboptimal results of TAVR with the current generation THVs are attributed to anatomical factors such as a lack of annular calcium, a large aortic annulus, and a dilated aortic root, posing challenges for device anchoring. TAVR with dedicated devices for AR, such as the JenaValve and the J-Valve, is rapidly evolving, with preliminary evidence suggesting higher procedural success rates and improved clinical outcomes during short-term follow-up. There is a significant unmet need for the development of transcatheter therapies with dedicated THVs for severe AR, and both the JenaValve and J-Valve systems are undergoing rigorous clinical trial testing before regulatory approval in the United States.

## Highlights

• Untreated clinically significant aortic regurgitation (AR) is associated with worse outcomes, including higher mortality.
• Although surgical aortic valve replacement is considered the treatment of choice for severe AR, a significant proportion of patients are not ideal surgical candidates due to underlying comorbidities, highlighting the significant unmet need for transcatheter options in this patient population.
• Transcatheter aortic valve replacement for severe AR with commercially available transcatheter heart valves is associated with suboptimal outcomes due to various anatomical factors and device-related challenges.
• Transcatheter aortic valve replacement for AR with dedicated devices such as the JenaValve and J-Valve is rapidly evolving, with preliminary data showing promising results with high procedural success rates and improved clinical outcomes.

## Introduction

Clinically significant (≥moderate) aortic regurgitation (AR) is common in the general population, with an estimated prevalence of ≥4% among patients ≥65 years of age.^1^ Degenerative aortic valve (AV) disease is the most common cause of AR, whereas endocarditis, rheumatic heart disease, bicuspid pathology, and aortic root disorders contribute to the rest. The pathophysiology of AR is complex and encompasses both pressure and volume overload. Chronic severe AR increases the left ventricular (LV) end-diastolic volume, leading to an increase in LV wall stress. If left untreated, LV remodeling ensues with eccentric hypertrophy and dilatation to accommodate the extra volume and maintain cardiac output. Diastolic and microvascular dysfunction, along with interstitial cellular changes, characterize this phase, and patients usually remain asymptomatic. However, LV compliance is reduced over time, causing elevated filling pressures and a decline in ejection fraction. This subsequent phase is characterized by symptoms of heart failure (HF) and eventually irreversible replacement fibrosis.^2^

Untreated severe AR is common and is associated with worse clinical outcomes, including higher mortality.^3^ Surgical AV replacement/repair (SAVR) is considered the gold standard for managing severe and symptomatic AR. Transcatheter AV replacement (TAVR) for pure AR is contraindicated (class III recommendation) in the most recent iteration of valve guidelines.^4^ However, the emergence of dedicated TAVR devices for pure AR has the potential to dramatically advance the field. This review aims to provide an overview of the evolution of TAVR as a potential alternative to SAVR in patients with pure AR, imaging challenges in diagnosing AR, and discuss current evidence of TAVR with commercial and dedicated transcatheter heart valve (THV) platforms and future trials in this rapidly advancing field.

### Imaging Challenges in Grading AR

Echocardiography is the first-line imaging modality to assess and quantify AR severity. AR quantification relies on a combination of qualitative, semiquantitative, and quantitative parameters (Table 1).^5^ However, there are several pitfalls to the multiparametric echocardiographic assessment of AR, including jet eccentricity, discordance, lack of hierarchy, and incompleteness.^6^ For example, the jet width to LV outflow tract (LVOT) diameter ratio underestimates AR in eccentric jets and is affected by LVOT diameter. Vena contracta, proximal flow convergence, effective regurgitant orifice area, and regurgitant volume (RV) are not reliable in the presence of multiple jets. Lastly, flow reversal in the proximal descending aorta is not reliable in elderly patients and is highly dependent on the compliance of the aorta. Although no single parameter is preferred or considered sufficient to assess the severity of AR, a combination of quantitative, semiquantitative, and qualitative methods is generally recommended.^6^

**Table 1:** Imaging criteria for severe AR

Echocardiographic parameters needed to diagnose severe AR	CMR criteria to diagnose severe AR
• Doppler jet width ≥65% of left ventricular outflow tract width • Regurgitant fraction ≥50% • Holodiastolic flow reversal in the proximal abdominal aorta • Effective regurgitant orifice area ≥0.3 cm^2^ • Vena contracta >0.6 cm	• Regurgitant volume >42 mL/beat∗ • Regurgitant fraction >33%∗ • Holodiastolic flow reversal with a minimum of 10mL/sec in the descending aorta in phase contrast images • Anatomic regurgitant orifice area >0.28 cm^2^∗

Abbreviations: AR, aortic regurgitation; CMR, cardiac magnetic resonance.

∗ These CMR thresholds were associated with the risk of progression to aortic valve replacement, although the cutoffs are currently not standardized.

Echocardiography is not only used for AR quantification, but also plays a key role in defining thresholds for intervention. The 2020 American College of Cardiology/American Heart Association valvular heart disease guidelines recommend surgery in asymptomatic patients with chronic, severe AR and LV ejection fraction (LVEF) >55% if 1) LV end-systolic dimension (LVESD) is >50 mm or LVESD index >25 mm/m^2^, 2) a progressive decline in LVEF over at least 3 sequential studies is observed to a low-normal range, or 3) there is severe LV dilatation with an LV end-diastolic diameter of >65 mm.^4^ However, recent data suggest worse clinical outcomes with a strategy of watchful waiting in patients with ≥ grade III AR and LVESD index 20-25 mm/m^2^, suggesting the ideal cutoff may be lower than previously recommended.^7^^,^^8^

Cardiac magnetic resonance (CMR) imaging is recommended as an adjunct imaging modality to assess AR in situations where echocardiography is inconclusive or there is a discrepancy between clinical examination and imaging findings. Interestingly, CMR-regurgitant fraction (RF) cutoff values for severe AR are consistently lower than those derived from echocardiography. Multiple studies have demonstrated a CMR-RF cutoff of 32%-35% to be associated with symptom progression and need for AV replacement.^9^, ^10^, ^11^, ^12^ CMR-assessed RV (CMR-RV) and CMR-RF are highly reproducible.^13^

### Management of Chronic AR

#### Surgical Management of AR

The optimal timing of intervention for AR is determined by symptoms, LVEF, and chamber dimensions.^4^ AV replacement (AVR) significantly improves the long-term survival in these patients.^7^^,^^14^^,^^15^ Evidence from the STS database suggests excellent outcomes in patients undergoing isolated SAVR for severe AR, with an operative mortality of 1.1%.^16^ With an increase in AR clinical stage (from C1 to D), there is a considerable increase in both operative mortality from 0.4 to 1.6% and major morbidity from 5.1 to 9.9%. Symptomatic AR is associated with a 2.3-fold increase in operative mortality. Similarly, LVEF <30% is associated with a 1.2-fold higher mortality rate than those with LVEF >30%. These findings suggest that earlier intervention, especially before symptom onset or development of adverse LV remodeling, may be warranted to optimize procedural outcomes. Despite excellent outcomes with SAVR, a significant proportion of patients with severe AR do not receive timely AVR due to advanced age, adverse LV remodeling, and underlying comorbidities, highlighting the need for transcatheter options. In the 2003 Euro Heart Survey, only 22% of severe AR patients with LVEF 30%-50% underwent SAVR, and among those with severe LV dysfunction (<30%), the proportion of patients receiving AVR was only 2.7%.^17^ TAVR with dedicated THVs can potentially fill this gap and improve outcomes.

### TAVR in AR

#### TAVR for AR with Commercial THV Devices

TAVR has revolutionized the management of aortic stenosis, with trials showing comparable results to SAVR across the spectrum of surgical risks.^18^, ^19^, ^20^, ^21^, ^22^, ^23^ However, TAVR for isolated pure AR has been associated with lower procedural success, increased procedural complications, and poor clinical outcomes.^24^^,^^25^

In a systematic review of 237 patients from 13 reports, device success ranged between 74 and 100%, with 7% of patients needing a second valve.^26^ A self-expanding valve (SEV) was used in 79%, and a balloon-expandable valve (BEV) was used in the rest. Thirty-day all-cause mortality was 7% and ≥moderate postprocedural AR was seen in 9% of patients. Similarly, in a 40-center international registry comprising 331 symptomatic patients undergoing TAVR for pure AR, Yoon et al.^24^ reported improved outcomes with newer-generation TAVR devices compared to the early-generation devices. Overall device success was 61.3% with early-generation THVs compared to 81.1% with the newer-generation devices, largely due to lower rates of second THV implantation (24.4 vs. 12.7%). Improvement in THV designs, operator experience, preprocedural and intraprocedural imaging guidance, and more appropriate patient selection might have contributed to this observation. Despite this, there were substantial procedural complications, and outcomes were suboptimal, with 18% pacemaker implantation, ∼10% ≥moderate postprocedural AR, and 24.1% all-cause mortality at 1 year.

**Figure 1:**
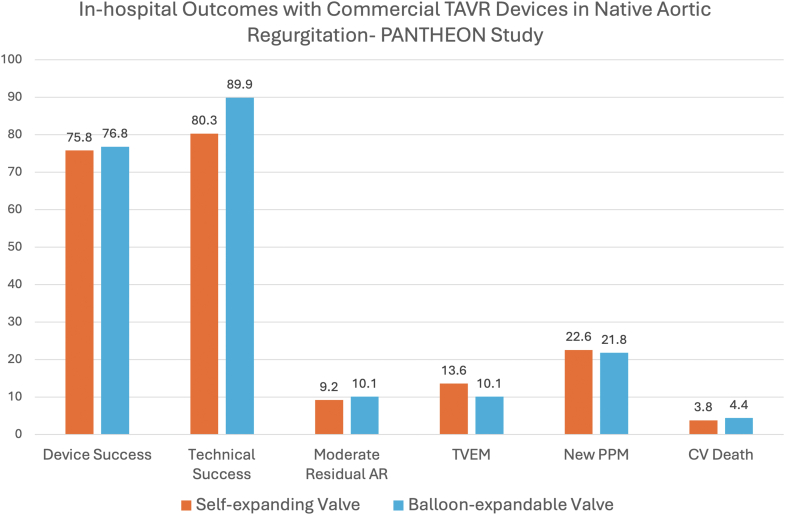
In-hospital outcomes after TAVR for AR with commercially available THVs in the PANTHEON study. No significant difference was noted in device and technical success rates, as well as clinical outcomes. Abbreviations: AR, aortic regurgitation; CV, cardiovascular; PPM, permanent pacemaker; TAVR, transcatheter aortic valve replacement; TVEM, transcatheter valve embolization or migration; THV, transcatheter heart valve.

The Performance of Currently Available Transcatheter Aortic Valve Platforms in Inoperable Patients With Pure Aortic Regurgitation of a Native Valve study retrospectively evaluated the outcomes of TAVR with current-generation BEVs and SEVs.^27^ In this study, a total of 201 patients were included. The overall technical and device success rates were 83.6 and 76.1%, respectively, and were similar between SEVs and BEVs (Figure 1). Moderate or greater residual AR was seen in 10% of patients. In-hospital mortality was high at 5%, and more than 1 in 5 patients required permanent pacemaker (PPM) implantation. THV migration/embolization rate was 12.4% in the whole cohort (13.6% for SEV and 10.1% for BEV). Almost all THV migration/embolization occurred during the index procedure, warranting implantation of a second THV. Compared to patients without, those with THV migration/embolization had worse outcomes at 1 year, with a composite endpoint incidence of 25.7 vs. 15.8%. Procedural and in-hospital outcomes were similar between the 2 valve designs.

Among Medicare beneficiaries who underwent TAVR with commercial THV devices or SAVR for pure AR between 2016 and 2019, Mentias et al.^28^ reported comparable in-hospital and short-term mortality between the 2 strategies. However, midterm to long-term outcomes were worse with TAVR than with SAVR. All-cause mortality, HF admissions, and need for redo-TAVR were higher in the TAVR group, likely due to older age, frailty, more comorbidities, and irreversible LV dysfunction, which is frequently seen in these patients.

#### Limitations of TAVR for AR with Commercial THV Devices

The suboptimal clinical outcomes reported with off-label use of commercial TAVR valves in patients with severe AR are, in part, a consequence of anatomical factors that make device anchoring difficult or impossible. THV migration/embolization may be associated with catastrophic complications, including mortality, need for a second THV, surgical conversion, and HF rehospitalizations.^27^ Factors associated with THV migration or embolization include device undersizing, lack of AV annular calcification, horizontal aorta, inadequate pacing, incorrect positioning due to inability to define the annular plane, postdilatation leading to device foreshortening, and suction effect from regurgitation into the LV.^27^ These anatomical factors can also result in inadequate sealing and paravalvular leak (PVL).

The concept of multiple anchoring zones from LVOT to the ascending aorta is currently being evaluated in the anatomical classification and dual anchoring theory to optimize the TAVR strategy for pure severe AR (AURORA), a prospective multicenter cohort study.^29^ The investigators propose a new morphological classification of the aortic root anatomy to guide TAVR in pure AR patients using a novel double-anchoring strategy. Based on a preliminary study, the investigators theorize that during TAVR for pure AR, the THV may be anchored at the LVOT, the aortic annulus, or the ascending aorta. For example, BEVs anchor at the LVOT and AV annulus levels, whereas SEVs exert radial force both at the annular level and the ascending aorta. The safety and efficacy of the double-anchoring concept require clinical validation in a larger cohort of patients.

No current-generation THV devices have received regulatory approval in the United States for use in pure isolated AR, and the suboptimal results with off-label use of commercial TAVR devices have prompted the development of dedicated devices.

#### TAVR for AR with Dedicated THV Devices—Device Description and Clinical Evidence

##### JenaValve

###### Design

**Figure 2:**
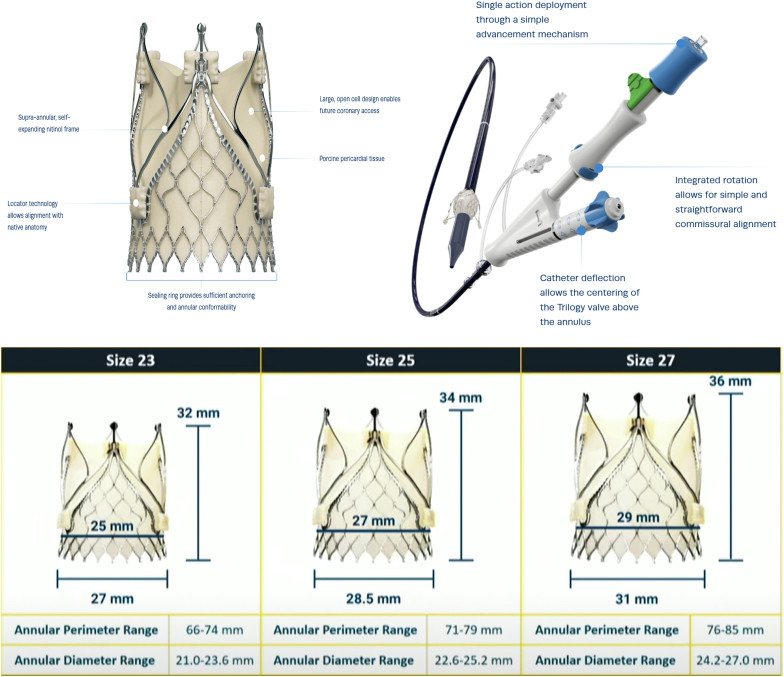
The JenaValve Trilogy transcatheter system (upper panel). The device consists of a self-expanding nitinol frame with supra-annular porcine pericardial leaflets. The 3 locators (angiographically visible) aid in anchoring the valve to the native AV leaflets and also help maintain commissural alignment. The large open cell design facilitates future coronary access. The valve delivery system consists of a 22F 65 cm long sheath. The JenaValve Trilogy system is available in 3 sizes (23, 25, and 27 mm) (bottom panel) and can treat AV annuli ranging from 66 to 85 mm in perimeter. Abbreviation: AV, aortic valve.

The JenaValve is a self-expanding transfemoral THV with supra-annular porcine pericardial leaflets and a nitinol frame (Figure 2). The device consists of 3 locators, which are used to align with the native AV cusps and anchor them during valve deployment. The locators can be rotated above the AV to ensure valve alignment with the native sinuses. As the valve expands, the locators clasp onto the native leaflets, providing an effective seal to prevent PVL and valve migration during deployment. The JenaValve Trilogy system is currently available in 3 sizes, 23, 25, and 27 mm, to treat AV annuli ranging from 66 to 90 mm in perimeter. The JenaValve Trilogy system has received the Conformité Européenne mark approval in Europe and is commercially available. Regulatory approval for the commercial use in the United States is expected in the last quarter of 2025.

###### Procedural steps

**Figure 3:**
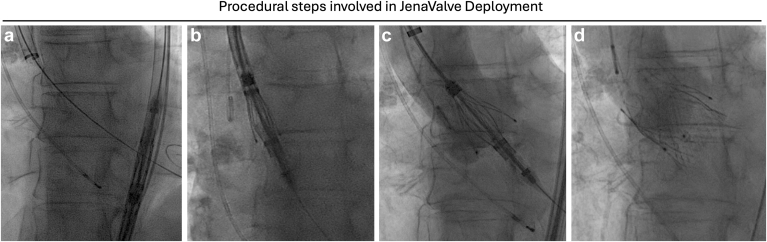
Procedural steps involved in JenaValve deployment. (a) The 22F 65 cm long sheath is advanced to the sinotubular junction. (b) The valve delivery system advanced through the sheath into the ascending aorta. (c) Locators are exposed and advanced to the AV cusps. The locator position is confirmed by aortic root angiography and transesophageal echocardiography. (d) Once positioning of locators and valve depth is confirmed, the valve is deployed. Abbreviation: AV, aortic valve.

1. Femoral artery access with placement of the 22F 65-cm sheath. The sheath is advanced to the sinotubular junction (Figure 3).
2. The sheath is retracted, which results in exposure of 3 valve locators, which are then advanced to the respective cusps. The locators can be rotated clockwise or counterclockwise if necessary.
3. Once the locators’ position is confirmed, the valve is deployed under rapid pacing by simply advancing the blue knob, which in turn releases the stent holder and allows for the nitinol frame to expand.

##### ALIGN-AR Trial and Early Commercial Experience

The ALIGN-AR trial was a prospective, multicenter, single-arm study that evaluated the safety and efficacy of the JenaValve Trilogy heart valve system in patients with moderate-to-severe or severe AR at high surgical risk.^30^ Between 2018 and 2022, 180 patients with symptomatic AR were enrolled in the trial. Bicuspid AV morphology was excluded. Technical success was achieved in 95% of patients. The primary efficacy endpoint of all-cause mortality at 1 year was 7.8%, achieving noninferiority (*p* noninferiority <0.0001) to an established objective performance goal. The primary safety endpoint, a nonhierarchical composite consisting of all-cause mortality, stroke, life-threatening or major bleeding, acute kidney injury (AKI) stage 2 to 3 or dialysis, major vascular complications, surgery or intervention related to the device (including coronary intervention), new PPM, and ≥ moderate residual AR at 30-days was seen in 27% of patients (*p* noninferiority <0.0001). The overall new PPM implantation rate was 24%, with a progressive reduction noted by tercile of enrollment ascribed to changes in the valve sizing algorithm as well as the depth of valve implantation (from 29% in the initial two-third of enrollees to 14% in the final one-third). Valve embolization was seen in 2 patients, and there were no cases of coronary occlusion or annular rupture. At 30 days, there were significant improvements in New York Heart Association (NYHA) functional class status, Kansas City Cardiomyopathy Questionnaire (KCCQ) overall score, and 6-minute walk test compared to baseline.

**Figure 4:**
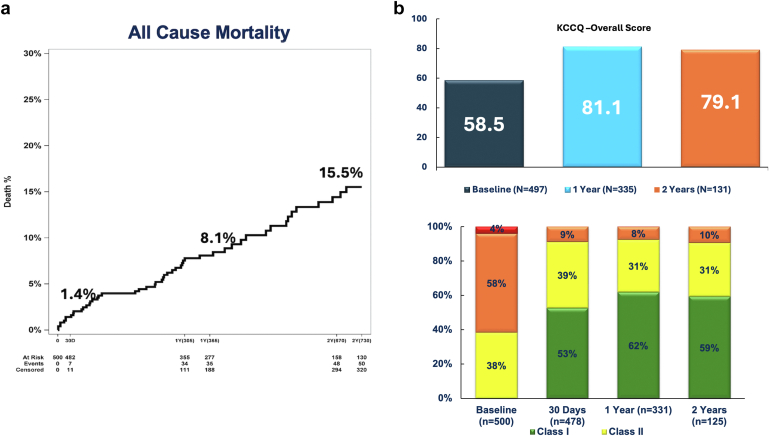
ALIGN-AR trial and continued access registry. (a) All-cause mortality at 1 year was observed in 8.1% of patients with approximately 50% being noncardiovascular (non-inferiority margin: 25%). There was also a significant improvement in (b) the KCCQ overall score and (c) New York Heart Association (NYHA) functional class 2 years after TAVR with JenaValve Trilogy System. Abbreviations: KCCQ, Kansas City Cardiomyopathy Questionnaire; TAVR, transcatheter aortic valve replacement.

The 2-year outcomes of this study were recently presented at the American College of Cardiology 2025 Annual Scientific Sessions.^31^ Among 500 patients who were enrolled in the investigational device exemption and continued access registry, the composite primary safety endpoint occurred in 26.2% at 30 days (Figure 4). The primary efficacy endpoint was seen in 8.1% of patients. Both endpoints met predefined noninferiority targets. In addition, echocardiographic data revealed favorable hemodynamics and reverse LV remodeling. The effective orifice area was 2.8 cm^2^ at 2 years. LV mass index, end-systolic volume index, and end-diastolic volume index decreased significantly compared to baseline. At 2 years, 95.7% of patients had PVL ≤ trace. The mean KCCQ overall summary score increased by 19.4 points. New PPM implantation varied by valve size and was 28.9% for 27-mm valves compared to 16.8% for 23-mm valves. These results suggest maintained safety and efficacy of the JenaValve Trilogy system at 2 years.

In the initial European commercial experience of the JenaValve in 58 consecutive patients with isolated symptomatic AR, Adam et al. reported 98% device success rates with no ≥moderate residual AR, no device migration/embolization, and no major vascular complications. All-cause mortality at 30 days was 1.7%, and new PPM implantation was 20%.^32^

##### J-Valve

###### Design

**Figure 5:**
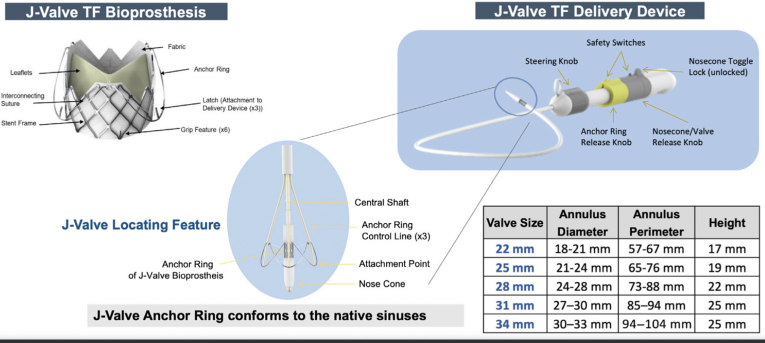
The J-Valve consists of porcine pericardial leaflets within a self-expanding frame and 3 U-shaped nitinol anchor rings. The anchor rings conform to the AV cusps and aid in anchoring the valve to the native leaflets. The J-Valve is available in 5 different sizes and can treat AV annuli ranging from 57 to 104 mm in perimeter. The delivery system consists of an 18F steerable catheter for precision valve deployment. Abbreviation: AV, aortic valve; TF, transfemoral.

J-Valve is a novel SEV with a low-profile Nitinol frame and bovine pericardial leaflets (Figure 5). The device consists of 3 U-shaped nitinol anchor rings that are designed to conform to the native AV sinuses and help anchor the AV leaflets to the inner valve frame. The design of the anchor rings also maintains commissural alignment during valve deployment. The anchors and the valve are actuated by 2 independent knobs on the catheter handle. The valve is available in 5 different sizes (22, 25, 28, 31, and 34 mm) and can treat a wide range of AV annular perimeters (upper limit perimeter of 104 mm) (Figure 3). The delivery catheter is steerable and flexible, which allows the treatment of complex aortic root anatomies, including horizontal aortas.

###### Procedural steps

**Figure 6:**
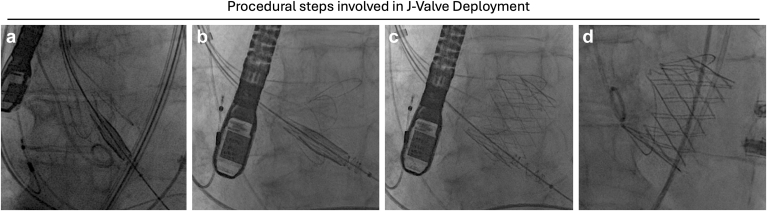
Procedural steps involved in J-Valve deployment. (a) After advancing the J-Valve delivery system into the ascending aorta, the U-shaped nitinol anchor rings are exposed at the level of the sinotubular junction. (b) The anchor rings are advanced to the respective aortic sinuses and confirmed by root angiography and/or transesophageal echocardiography. (c) J-Valve deployed, note the anchor rings are still attached to the control lines. (d) Final fluoroscopy image showing fully deployed J-Valve after release of control lines.

1. Femoral artery access and placement of a 20F (22-28 mm valves) or 22F (31 and 34 mm valves) Gore DrySeal or 16 Fr e-sheath (Figure 6).
2. J-Valve advanced into the ascending aorta, exposure of U-shaped anchor rings at the level of the sinotubular junction (2 pigtails above the annular plane)
3. The anchor rings are then advanced to the nadir of the respective sinuses by advancing the yellow anchor ring release knob. This is confirmed on fluoroscopy and transesophageal echocardiogram.
4. Once the position of the anchor rings is confirmed and deemed satisfactory, the valve stent frame is positioned with its superior edge of the frame (outflow) at the level of the superior edge of the anchor rings. The gray nosecone/valve release knob is then turned clockwise to deploy the valve.
5. After valve deployment, the anchor rings' control lines are released.

##### Compassionate Use Experience and Early Feasibility Study

The very early experience with this device occurred in China using transapical access in 6 patients who were at a high or prohibitive risk for surgery.^33^ The delivery sheath size used for the transapical approach was 27F. In its current iteration, the device is delivered via transfemoral access through a 16 Fr expandable sheath (Edwards e-sheath) or a 20-22 Fr DrySeal.

In the J-Valve North American compassionate use experience, 27 patients with severe AR at a high surgical risk and with NYHA functional class III/IV were included between 2018 and 2022.^34^ The procedural success rate in the overall cohort was 81%, which was attributed to a suboptimal valve design and liberal case selection. In 2 cases, there was inadequate device anchoring due to AV leaflet prolapse, prompting the exclusion of patients with this anatomy from future enrollment. Valve migration requiring implantation of a second valve was observed in 3 patients. Subsequent modifications to the THV design and enrollment criteria resulted in a 100% device success rate in the last 15 consecutive patients. There was 1 death, a 13% pacemaker implantation rate, and no cases of residual AR ≥ mild at 30 days. The average mean AV gradients were <10 mm Hg with an effective orifice area of >2 cm^2^.

In the J-Valve early feasibility study, 15 high-risk patients with severe symptomatic AR were prospectively enrolled.^35^ Patients with bicuspid AV, dilated aortic root >5 cm, mixed AV disease, and LVEF <25% were excluded. Procedural success was 93.5%, with 1 patient requiring conversion to surgery due to the inability to release the anchor rings post valve deployment. There were no cases of intraprocedural mortality or valve embolization. At 30 days, there were no reports of cardiovascular mortality, HF hospitalization, strokes, new pacemakers, or device-related interventions. Echocardiographic analysis showed no ≥ mild residual AR, low single-digit transvalvular gradients, and a mean effective orifice area of 2.9 ± 0.68 cm^2^. In addition, there was evidence of reverse LV remodeling with a decrease in LV end-diastolic dimensions and volumes. This study demonstrated the safety and efficacy of the J-Valve system in treating AR with an excellent hemodynamic profile at 30 days. Notably, nearly 40% of patients enrolled in the early feasibility study had an AV annulus perimeter >90 mm, which would have been exclusionary for the JenaValve Trilogy system.

#### Comparison of TAVR with Dedicated Devices vs. Off-Label Commercial THVs

**Figure 7:**
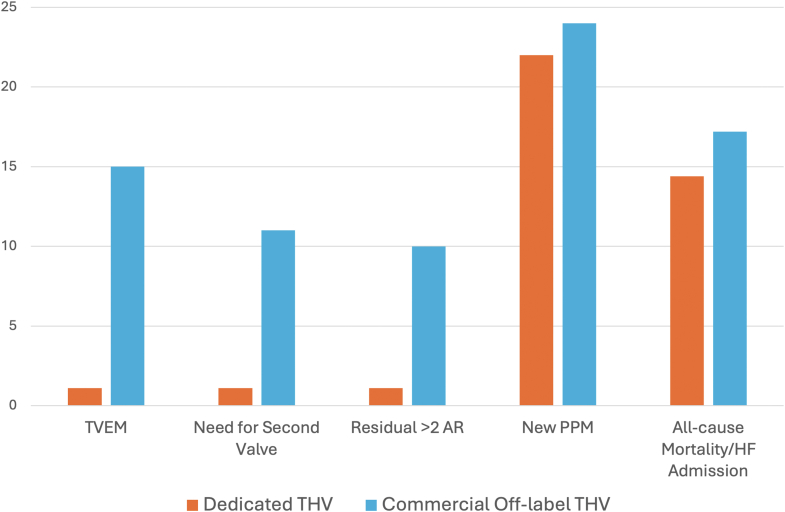
PURPOSE study. TAVR with dedicated THVs was associated with lower rates of TVEM, residual AR but with similar rates of new PPM and all-cause mortality/HF admissions compared to commercial nondedicated THVs. Abbreviations: AR, aortic regurgitation; HF, heart failure; PPM, permanent pacemaker; TAVR, transcatheter aortic valve replacement; THV, transcatheter heart valve; TVEM, transcatheter valve embolization or migration.

There are no prospective, randomized studies comparing the safety and efficacy of dedicated vs. off-label commercial devices for the treatment of native AR. However, important lessons can be learned from observational registries. The PURPOSE study was a multicenter, retrospective registry involving patients from 18 high-volume centers in Europe and the United States.^36^ Overall, 256 patients were included, of which 168 underwent TAVR with commercial THVs and 88 with JenaValve. Compared to commercial THVs, TAVR with JenaValve was associated with superior technical (98 vs. 81%) and device success rates (95 vs. 73%), significantly lower device embolization (1.1 vs. 15%), and moderate residual AR (1.1 vs. 10%). New PPM implantation rates were similar (Figure 7). Despite the superior performance of the JenaValve, there were no significant differences in all-cause mortality or HF hospitalizations at 1 year.

In a recent meta-analysis of 34 studies involving 2162 patients with native AR, Samimi et al. showed superior outcomes with dedicated THV devices compared to commercially available THVs.^25^ TAVR with dedicated THVs was associated with higher device success rates and lower mortality, residual AR, reintervention, new PPM implantation, and valve embolization/migration. Time-sensitive analysis, after exclusion of studies performed before 2016, showed worse outcomes at 30 days with off-label use of commercial THVs with respect to all-cause mortality, reintervention, and valve embolization/migration. Interestingly, the analysis also evaluated outcomes between JenaValve (transfemoral and transapical) and J-Valve (transapical). Although there was no difference in all-cause mortality, device success, reintervention, stroke, and major vascular complications, the new PPM implantation rate was significantly higher in the JenaValve group. This is likely a reflection of the shorter J-Valve stent frame, with an attendant lower profile in the LVOT, and lower radial force. Residual AR ≥ moderate was more frequently seen in the J-Valve group. To date, this is the only study indirectly evaluating outcomes by comparing the 2 dedicated THV devices, although the results should be interpreted with caution given the variability in access, device iterations, and operator experience.

**Figure 8:**
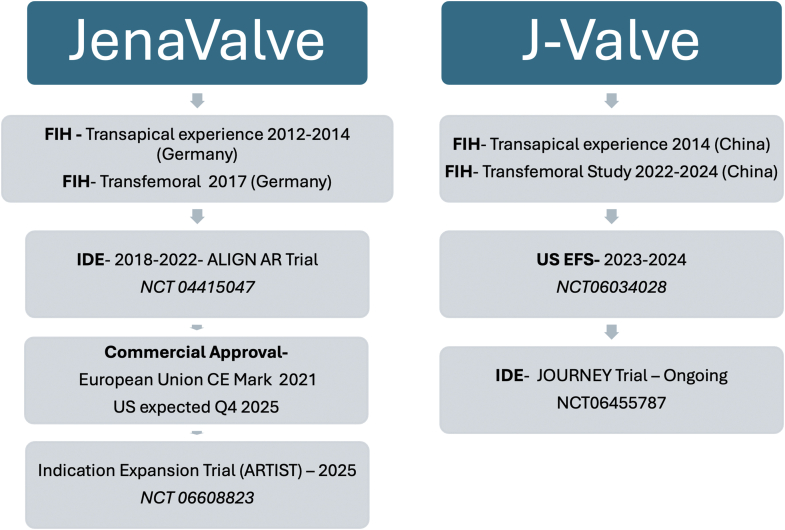
Timeline of significant clinical milestones in clinical development pathways for dedicated TAVR devices for native aortic regurgitation. Abbreviations: CE, Conformité Européenne; EFS, early feasibility study; FIH, first in human; IDE, investigational device exemption.

Both valves are under rigorous clinical trial testing in the United States and have yet to receive regulatory approval. A detailed timeline from the first in-human experience to current investigational device exemption trials for both dedicated devices is presented in Figure 8.

##### Special Subgroups: AR in Patients with LV Assist Devices

Moderate or more AR is frequently seen in patients with LV assist devices (LVAD). Within the first year after LVAD implantation, the incidence of ≥moderate AR is about 14% to 24% and is expected to increase thereafter.^37^^,^^38^

In patients with LVAD, significant AR creates a circulatory loop, which results in elevated LV end-diastolic pressures and reduced organ perfusion. Mechanisms of AR in LVAD include increased shear stress of AV leaflets, commissural fusion, and alterations in the aortic root/sinuses. Surgical reintervention is considered high or prohibitive risk in these patients. Moreover, LVAD patients have been historically excluded from clinical trials. TAVR with commercially available THV devices has been performed with variable success in isolated case reports and small case series.^39^^,^^40^ Patients with LVAD and AR face similar challenges to those with pure native AR during TAVR due to a lack of AV calcium to anchor the THV, and the frequent presence of a dilated aortic root/sinuses and LVOT, risking valve embolization/migration, often requiring a second THV implantation.

Evidence in support of TAVR with dedicated THV devices for AR in LVAD patients is limited to case reports at present, but ongoing studies are expected to shed light on this approach.^41^^,^^42^ The JENA-VAD is a prospective, multicenter, single-arm registry designed to assess the safety and efficacy of TAVR with the JenaValve Trilogy heart valve system in LVAD patients. The study will include patients with continuous flow LVAD, clinically significant AR, and NYHA functional class III/IV symptoms who are at high risk for SAVR. Primary outcomes assessed at 30 days include new PPM implantation, device success, PVL, residual AR, freedom from surgery or reintervention, any stroke, AKI, vascular complications, and all-cause mortality.

#### Ongoing and Future Trials

The Aortic Regurgitation Trial Investigating Surgery versus Trilogy (ARTIST) study (NCT06608823) is a randomized controlled trial comparing the safety and efficacy of the JenaValve Trilogy transcatheter system with SAVR in non–high-risk patients with symptomatic AR. The study aims to demonstrate noninferiority of TAVR with JenaValve compared to SAVR. The trial is planning to enroll nearly 1000 patients and has a composite primary outcome of all-cause mortality, any stroke, and unplanned cardiac rehospitalization at 12 months.

The J-Valve to Treat Aortic Regurgitation Via Transcatheter Therapy pivotal trial (NCT06455787) is a prospective, single-arm, multicenter study designed to assess the safety and efficacy of the J-Valve system in patients with severe symptomatic AR and high surgical risks. Nearly 200 patients will be enrolled at 35 sites in the United States, Canada, Europe, and Japan. Primary endpoints include all-cause mortality at 1 year and a composite of all-cause death, any stroke, major bleeding, AKI, new PPM, major vascular complications, and repeat intervention at 30 days.

#### Other Devices Currently Under Development Outside the United States

The Hanchor Valve system (INT Medical, Shanghai, China) is a novel transfemoral BEV with bovine pericardial leaflets, cobalt-chromium frame, and nitinol anchor elements that are designed for leaflet clamping. The valve was evaluated in the Multi-Center Trial of Hanchor Valve for Treating Patients With Severe Pure Native Aortic Regurgitation (HAVE AR) trial, which was a prospective, multicenter, single-arm study that enrolled 128 patients with severe AR between January 2023 and November 2023 at 13 centers in China.^43^ Procedural success was noted in 96% of the patients. There were 3 instances of valve migration (2%), with 2 needing a second valve and 1 needing surgical conversion. At 30 days, new PPM implantation rate was 12% and all-cause mortality was 2%. Significant improvements in NYHA functional class were noted, and echocardiographic assessment showed evidence of LV reverse remodeling.

MyVal (Meril Life Sciences, Vapi, India) is a BEV with a unique design that allows TAVR in patients with a large AV annulus (up to 840 mm^2^), a feature commonly seen in pure AR patients. Although the device received Conformité Européenne mark approval for the use in severe aortic stenosis, robust evidence in pure AR is currently lacking. In an international, multicenter, observational study, Sanchez-Luna et al.^44^ enrolled 113 consecutive patients with severe symptomatic AR undergoing TAVR with the MyVal THV (37565470).^44^ The procedural success rate was 92% with a 3.5% incidence of valve embolization needing implantation of a second THV. Moderate-to-severe residual AR was seen in 9% of patients, and the rate of new PPM was 13.4%. There are no studies comparing MyVal with other commercially available or dedicated THVs for AR.

## Conclusion

There is a significant unmet clinical need for transcatheter therapies in patients with severe, symptomatic native AR. Dedicated TAVR devices are currently under investigation in the United States. and are likely to become clinically available in the near future following years of rigorous clinical trial testing. Importantly, clinical trials for dedicated TAVR devices include imaging substudies (cardiac magnetic resonance imaging) and inclusion of special populations (LVAD), both of which are expected to inform societal guidelines.

## Funding

The authors have no funding to report.

## Disclosure Statement

Dean Kereiakes: Consultant for Edwards Lifesciences, study Chair JOURNEY trial.

Santiago Garcia: Consultant, proctor for Edwards Lifesciences, National PI JOURNEY trial.

The other authors had no conflicts to declare.
